# The emotional intelligence of today’s parents – influences on parenting style and parental competence

**DOI:** 10.3389/fpubh.2023.1120994

**Published:** 2023-07-04

**Authors:** Andreea Șițoiu, Georgeta Pânișoară

**Affiliations:** ^1^Doctoral School “Psychology and Educational Sciences”, University of Bucharest, Bucharest, Romania; ^2^Department of Psychology and Educational Sciences, Faculty of Psychology and Educational Sciences, Transilvania University, Brasov, Romania; ^3^Faculty of Psychology and Educational Sciences, University of Bucharest, Bucharest, Romania

**Keywords:** emotional intelligence, parental competence, parenting style, self-esteem, Barnum effect

## Abstract

The emotional intelligence of parents and parental competence become two premises of effective parenting. This study contributes to the understanding of how parents’ emotional intelligence exerts its effect on both their parenting style and parenting competence. The present research also sought to identify the factors that determine the level of parental competence. The research method used is the questionnaire-based survey. The research sample, represented by 610 respondents, was determined by the sampling strategy based on convenience, respectively by the snowball method. The results indicated that the emotional intelligence of parents is associated with an increased level of parental competence (r = 0.24, *p* < 0.001), and 15% of the variability of parental competence is determined by the level of parental emotional intelligence (R = 0.38, *p* < 0.00, R^2^ = 0.15). It was demonstrated that 6% of the variability of parental competence is determined by the level of parents’ self-esteem (R = 0.24, *p* < 0.001, R^2^ = 0.06). The emotional intelligence and self-esteem of the parents contribute to the development of a high level of parental competence, while the level of education of the respondents according to this research partially influences the development of parental competence. Despite the fact that a high educational level of parents is assumed to give them access to quality information, the Barnum effect is experienced regardless of educational level. According to the field of parenting, the Barnum effect refers to consulting non-scientifically validated sources and trusting information that responds to parents’ wishes, but not to children’s educational needs.These results highlight the role of parents’ emotional intelligence on the growth and education of children, but also the usefulness of training programs in the field of parenting with the aim of training parents in the necessary skills for effective parenting.

## 1. Introduction

Parental education is considered an important pillar of society because it has the potential to mitigate or even diminish the negative effects of some factors that can determine the social exclusion of the child, such as: poverty, belonging to a single-parent family, the minimum level of parental education (1). The purpose of parenting education is to improve the conditions of child rearing and to provide support to parents in managing both child and parental behaviors (2).

Parental education involves a complex process often being avoided as a result of parents’ lack of resources to get involved in this process or the limited availability of time due to the multiple responsibilities associated with parenthood (3). found that as adults experience parenting anxiety, children experience higher rates of academic burnout.

Emotional intelligence is a component of social intelligence, representing a person’s ability to monitor their own feelings and emotions, respectively to differentiate them (4). This type of intelligence facilitates a person’s adaptation to stressful situations (5). According to Goleman (6), emotional intelligence has the following branches: “self-awareness, social awareness, managing one’s own emotions, managing relationships.”

A scientific approach to emotional intelligence considers the training of emotional skills, which lead to living a fulfilled and productive life (7), since emotional intelligence offers visible results in terms of the quality of social relationships, but also the development of some prosocial behaviors (8).

In agreement with Baumrind (9), the parenting style includes a series of practices and attitudes on the part of the parent related to his/her child, delineating three types of parenting: authoritarian, authoritative and permissive. These styles follow two dimensions of parenting, authority and affection. The first dimension, authority or exigency, refers to the parent’s level of control over his child’s behaviors by establishing limits, rational standards. The second dimension, affection or responsiveness, considers how the parent expresses love towards the child and the parent’s flexibility to accept the child’s viewpoints (10).

Regarding parental competence, following the research carried out by Glăveanu (11), a factorial model of it was identified, which includes the following factors: knowledge, discipline, time management, emotional support and crisis management. This type of competence represents a complex system of skills intended to support adults in fulfilling their parental responsibilities, respectively in preventing or dealing with difficult situations that may arise in the child’s education and that require management for the purpose of his development (12).

Parental competence evolves as a result of the permanent education to which the parent is subjected throughout his life in a society focused on knowledge, thus improving the quality of family life (13). It mediates parents’ reflective functioning, which refers to their ability to reflect on their children’s mental states and children’s emotional adjustment (14).

Based on the aforementioned aspects of the literature, this research tested the link between parents’ emotional intelligence and parenting style, as well as the link between emotional intelligence and parental competence. At the same time, parental competence was analyzed in relation to the level of parents’self-esteem, but also in relation to its determining factors.

### 1.1. Research aim and research hypotheses

The variables on which the entire study was conducted were the following: emotional intelligence, parenting style, parenting competence, parental self-esteem, and the Barnum effect. In order to test certain hypotheses, the variable referring to the educational level of the parents was also included. Starting from the previously mentioned variables, the purpose of the research was to establish the influences that parents’ emotional intelligence has on the parenting style adopted by them, but also on the level of parental competence. A second aim of the research was to identify the factors that determine the level of parents’ parental competence.

The research was based on the following objectives:

O1: Identifying the influence of parents’ emotional intelligence on the level of parental competence.

O2: Determining how the level of parental competence varies according to parents’ self-esteem.

O3: Identifying the factors that determine the level of parental competence.

O4: Identifying the role that parents’ self-esteem has on the relationship between emotional intelligence and parental competence.

O5: Identifying how parental competence varies by parents’ educational level.

O6: Identifying the link between parents’ emotional intelligence and parenting style.

O7: Identifying the role of the Barnum effect on parental competence.

Based on the aforementioned objectives, the following research hypotheses were formulated:

*H1*: As the level of emotional intelligence of parents increases, parental competence develops.

*H2*: Along with the increase in self-esteem of parents, the level of parental competence varies in a positive sense.

*H3*: Parental competence is determined by factors such as parents' emotional intelligence and self-esteem.

*H4*: The relationship between emotional intelligence and parental competence is mediated by the level of parents' self-esteem.

*H5*: Parental competence varies according to parents' educational level.

*H6*: As the level of parents' emotional intelligence increases, the preference for the authoritative parenting style increases.

*H7*: As the parent experiences the Barnum effect at a high level, the level of parental competence decreases.

## 2. Literature review

### 2.1. Emotional intelligence in parenting

Parents are often faced with the existence of inappropriate behaviors of the child, which can be the source of conflicts in many families. Since emotions specific to a conflict can be difficult to manage for most parents, at the same time influencing the appearance of the feeling of personal inadequacy or disappointment towards the child, the importance of emotional intelligence in order to ensure quality parenting even in tense situations is noted (15).

Emotional intelligence is closely interdependent with the education of feelings, which is why, including in the field of parental education, this type of intelligence requires more and more valorization (16). Goleman (17) defines emotional intelligence as the human ability to self-motivate and persevere in the face of stressful situations. This intelligence is also associated with a person’s resistance to controlling their impulses and delaying gratification.

After extensive studies conducted over 25 years, researcher Reuven Bar-On established the defining components of emotional intelligence as follows: intrapersonal aspect, interpersonal aspect, adaptability, stress control, general mood (18).

This intelligence is associated with people’s resistance to challenging life experiences, such as raising a child, experiences that they later integrate into new ways of acting (19). In a systematic review and meta-analysis, researcher Vega et al. (20) demonstrated that increased levels of emotional intelligence are associated with decreased aggressive behaviors that may occur as a result of stressful situations experienced by parents.

Personality traits such as: openness to experience, conscientiousness, extraversion, agreeableness are associated with the development of emotional intelligence (21) and implicitly with the formation of effective interpersonal and intrapersonal relationships (22), respectively with the ability to resolve conflicts (23).Therefore, this type of intelligence is found to become a predictor of individual psychological well-being (24).

In the absence of developing emotional intelligence, parents can experience what Goleman calls emotional overstimulation, a phenomenon in which they act and say things they would not have thought of if they were relaxed. These behaviors are associated with difficulties in children managing negative emotions such as anger (25). The acquisition of emotional intelligence in the case of parents is associated on the other hand with what (26) call “Sensitivity to context” referring to their ability to regulate their emotional responses according to the context in which they are.

In the case of parents, emotional intelligence becomes particularly important because it has been found that it alleviates their tendency towards perfectionism in the parenting process, which further leads to mental exhaustion (27). At the same time, the emotional intelligence makes parenting practices more efficient (28), having positive effects on the child’s development from all points of view. From the perspective of parental education, reducing stress as a result of parents’ emotional intelligence contributes to children’s socio-emotional development (29).

In a study that aimed to identify the roles of the intrapersonal and interpersonal dimensions of emotional intelligence in the parenting process, it was demonstrated that the intrapersonal dimension of this intelligence is associated with a decrease in the level of parental exhaustion (30).

### 2.2. Parenting style

From the perspective of researchers such as Baumrind (31), Maccoby and Martin (32), parenting style contributes to the study of child development, representing a combination of parental practices used to raise and educate him.

Parenting dimensions according to Baumrind (33) and Maccoby and Martin (32) cited in Zheng et al. (34) refer to responsiveness and demandingness. The first dimension is operationalized in terms such as respecting the child’s individuality, providing opportunities for the child to regulate his behavior and assert himself. Other behaviors specific to the responsive parent are supporting the child and adapting to the needs and requirements specific to the developmental stage. The second dimension refers to the requirement that must take care of him, which demonstrates the pressure exerted on the child in order to integrate into the family, the request of requirements that involve his early maturity, the making of efforts to discipline the child according to some high standards. Parental demand is also correlated with the parent’s desire to confront his own child at the expense of the effective relationship with him.

The reference model regarding parenting styles is represented by the two-dimensional model developed by Maccoby and Martin, a model that presents empirical evidence regarding parenting styles and child adaptation. According to this model, there are two dimensions through which parents relate to children, namely warmth and severity (35–42). Parental warmth is represented by the measurement in which parents offer children love, acceptance, approval, support, but also availability for dialogue (35), while parental severity is highlighted by the measurement in which parents use discipline in education children, monitoring their behavior, setting limits and at the same time maintaining his authority over them (36).

Based on the two dimensions, four parenting styles were identified: the authoritarian parenting style based on severity in the absence of warmth, the authoritative parenting style based on the balance between severity and parental warmth, the permissive/indulgent parenting style represented by the presence of parental warmth in the absence of strictness and the parental style careless which is characterized by the absence of both dimensions, warmth, respectively parental severity (35–37, 42–45).

Authoritative parenting style has been associated with stimulating proactive behaviors among children by improving the level of self-esteem and growth mindset (46), but also with the acquisition of their emotional autonomy (47). Parents following this style managed to find a balance between authority, flexibility and gentleness in the parenting process, while also demonstrating empathy towards children. Empathy felt by children whose parents chose to approach authoritative education is associated with reduced delinquency during adolescence (48).

In agreement with Shahsavari (49) the authoritative parenting style encountered in the specialized literature and in the form of the concept of democratic or authoritative parenting style is defined by showing a balance between exercise control over children and emotional support and maintaining an optimal level in terms of the child’s independence. Parental control from the perspective of this parental style can be operationalized in the form of the concept of the child and ensuring the optimal conditions in which it can develop and not the manifestation of an attitude of superiority towards it. Parents following this parenting style cause their children to be free to think, while also giving them opportunities to develop some skills.

Authoritarian parenting style has in mind high expectations of parents from children, emphasizing children’s compliance with rules at the expense of democratic discussions with them. Also, this parenting style is based on discipline and strict punishments in order to follow the rules, being also associated with an increased risk of child maltreatment (50, 51), but also with depressive symptoms among them (52).

The despotic or authoritarian parenting style is characterized by monitoring and evaluating children’s behavior according to certain standards, having high expectations from them and demonstrating a low level of acceptance of their attitudes and behaviors. A consequence of parents’ excessive authority over children’s development is mental and emotional instability, diminishing the possibility of experiencing well-being [(53) cited in (49)].

Parental authority is associated with children’s extreme concern for the mistakes of others and the imposition of high self-standards (54). Authoritarian parenting style causes externalizing behaviors such as aggression, delinquency, hyperactivity and internalizing behaviors such as social isolation, anxiety and suicidal behavior among children (55).

The permissive parenting style is associated with a high level of atypical behavior of children, but at the same time with generalized anxiety among them (56), respectively with the decrease of the feeling of academic self-efficacy (57). Those children who perceive their parents as permissive face during their life a decrease in the process of personal growth (58) and a reduced tendency to offer help to those around them in different situations (60). At the same time, the permissiveness of the parents in the parenting process is also noted by the absence of consequences when the rules are broken, i.e., giving the child the opportunity to make his own decisions despite the fact that he is not ready for this in all situations (61).

Parents following a permissive style of raising and educating their child do not possess the necessary skills to guide and regulate his behavior from early childhood. A consequence of this parenting style is the lack of optimal moral development and the formation of values during youth. At the same time, parental permissiveness is also associated with negative influences on children’s cognitive development, they face immaturity, decision-making in the presence of impulsiveness, respectively rebellion (59) cited in (49).

The negligent parenting style is defined by a low level of control, acceptance and receptivity in the parent–child relationship, failing to be optimally involved in its growth and development. The consequences of parental neglect are highlighted by the reduced experience of some moments of parent–child affection, but also by ridicule [(62) cited in (49)].

Kotchick et al. (63) respectively Cowan et al. (64) cited in Vafaeenejad et al. (65) concluded from the studies carried out that the attachment style of the parent, but also the family conditions in the past such as the stress experienced and the supportive relationships developed within the frame. Family influences the parenting style. The secure attachment of adults in agreement with the family of origin is transposed in the form of authority style with the acquisition of parent status, being more responsive to children and establishing a healthy relationship with them. In contrast to the secure attachment, the attachment based on absence or anxiety developed by the adult within the families of origin can face an authoritarian parenting style being dominated by anger and choosing to show a distant attitude towards the child. All these behaviors resulting from the acquired attachment style can have long-term consequences in terms of the effectiveness of interpersonal relationships, respectively the mental health of parents and children alike.

Maccoby and Martin’s two-dimensional model has led to a series of debates aimed at identifying the best parenting style for optimal child development and adjustment. Classic studies on parenting carried out in the Anglo-Saxon context demonstrated that the child’s psychosocial adaptation is largely influenced by authoritarian parenting (36, 39, 66, 67).

Authoritative parenting style was not found to be the most appropriate in child adjustment following studies conducted in ethnic groups in the United States, such as Chinese-Americans (68), African-Americans (69) and of some studies started in Arab societies (70). In their case, it was concluded that the authoritarian parenting style causes the children to adapt best to the society in which they live.

In the case of European and Latin American countries, it has been demonstrated that parental indulgence largely determines the child’s adaptation in society (37, 38, 41, 44) and long-term (35, 45, 71–74).

### 2.3. Parental competence

The term parental competence includes a series of many competences such as: communication, conflict resolution, negotiation, self-esteem development skills, but also social, motivational, cognitive and normative skills (75). This competence is associated with an involved parenting according to which it is important to protect the child by “ensuring protection in physical or psychological conditions of real danger, which represents a concrete attack on the physical or mental integrity of the child and on his well-being” (78, p.28). This is also associated with the balance that parents manage to establish between the children’s desire for autonomy and the need to provide them with guidance at all times. They succeed by combining gentleness with moderate firmness to make the child responsible (79).

The need to develop parental competence among adults derives from the multitude of challenges that they have to face in the process of educating the child (76).

Parental competence involves practices that are based on love, behavior modeling, intentional learning, and discipline (77). As a result of these skills and practices used, a family climate is established defined by the sense of belonging, the creation of trusting relationships, the development of psychological safety as well as values regarding the management of emotions (80). The parent who fulfills this competence promotes practices that take into account the child’s physical health and safety, practices that contribute to the child’s cognitive, emotional and social development, but also practices that take into account the optimal organization of the environment in which the child lives, of routines as well as the disciplinary approach based on positivism (81).

A competent parent approaches behaviors by which he finds out in detail what is happening with the child, what he feels, being with him and not against him; accepts the child’s feelings, even if he does not understand them very well at the initial stage of the critical situation, but acceptance is an essential behavior for later understanding; abandons the idea that in a conflict the parent must be right and replaces this idea with strategies to calm the child and redirect his attention (82); uses active listening by paraphrasing the child’s words so that he realizes that the parent has understood his situation; guides the child in solving the critical situation and does not take responsibility as a parent for solving the situation (83).

Regardless of the type of family the child belongs to, the most important aspect remains the management of all factors that can affect its evolution, and this management becomes all the more effective as the level of parental competence increases (86).

### 2.4. Parents’ self-esteem

Self-esteem is embodied in the way a person thinks about himself, about the values, the abilities he possesses (87), therefore this concept is associated with self-awareness (88), but also with general well-being (89).

People with high self-esteem experience the feeling of security in their own abilities and constructive pride in the successes achieved, while people with low self-esteem avoid externalizing their feelings, do not recognize their abilities, showing insecurity (90). For these reasons, self-esteem becomes an essential construct for understanding a person’s well-being and success (84).

The constant feeling of dignity, but also the ability to solve problems competently represent two attributes of the concept of self-esteem (85). People with a high level of self-esteem are self-confident and self-directed towards success (91), while low self-esteem is associated with the inability to manage various problems and one’s own existence, as well as the lack of energy (92), tending to procrastinate (93) and experiencing social anxiety (94, 95). High self-esteem is associated with feeling confident when a person wants to try new things, but also when faced with challenges (96).

In the case of adults, the way they perceive those around them, or the way they think they are perceived by those around them, influences self-esteem (97).

From a parenting perspective, parents’ self-esteem can have effects on children’s self-esteem as adults. It has been proven that a low self-esteem of parents influenced the formation of low self-esteem in the case of children reaching adulthood (98).

### 2.5. The Barnum effect in parenting

The Barnum effect, also often found in the field of parenting, involves recognizing oneself in general statements (99), ambiguous as being descriptive of one’s own personality (100) especially when those statements offer valid perspectives (101). This effect can also be found under the name of the Forer effect as a result of the one who first demonstrated that individuals willingly endorse universally valid statements as meaningful for the situation in which they find themselves (102). The same author found that individuals tend to accept false statements about themselves as long as they find them flattering and positive (103).

In agreement with the field of parenting, the Barnum effect is experienced in situations where parents are informed about the child’s upbringing and education from non-scientifically validated sources, supported by people without expertise in the field or with minimal training in this regard. As a result of the Barnum effect, parents’ perception of parenting is distorted. They believe that the parental practices identified in different sources are applied regardless of the specifics of the child, not paying the necessary attention to the child’s needs. In this situation, the parent’s action strategy for educating his child is not personalized. Experiencing the Barnum effect by parents can have negative consequences for child development as a result of existing limitations in parenting practices that are not based on scientific information (104).

## 3. Materials and methods

### 3.1. Participants and procedure

The sampling strategies used were convenience and snowball sampling.

The research sample consists of 610 respondents, 530 female, representing 87% of the total number of respondents, and 80 male, representing 13% of the total number of respondents. From the perspective of the age range of the research participants, 6 of them are under 20 years old (1%), 67 respondents are between 21 and 30 years old (11%), 205 respondents are between 31 and 40 years old (34%). The majority category of respondents from the perspective of age are those who fall between 41 and 50 years old, numbering 216 (35%), and the last category, representing the age of over 50, includes 116 respondents (19%).

From the point of view of educational level, the majority category of respondents is represented by those who completed university studies, in number 271 (44%), followed by those who completed post-graduate studies, in number 185 (30%) and by those who graduated from high school, in number 102 (17%).

The implementation of the research was carried out with the permission of the Ethics and Academic Integrity Commission of the University of Bucharest. Data collection was carried out between March 2022 and May 10, 2022, and the research tool was available on the Google Forms platform, with respondents having the opportunity to access it via a link. The data specific to each variable were collected in the Google forms platform, processed in an Excel database and later transferred to the Jamovi (105) statistical program. Another statistics program used was Jasp (106).

### 3.2. Measures

The research variables were parents’ emotional intelligence, parenting style, parental competence, parents’ self-esteem, the Barnum effect, and parents’ educational level.

The level of emotional intelligence and implicit empathy of parents was measured with the instrument ESCQ-45 - Emotional Skills and Competence Questionnaire, Vladimir Taksic (107).The questionnaire consists of 45 items distributed in three scales: The Perception and Development of Emotions scale, the Expression and Labeling of Emotions scale and the Management and Regulation of Emotions scale.The responses being provided on a five-step Likert scale, where 1 – Never and 5 – Always. Examples of items: Unpleasant experiences teach me how not to act in the future, When I see how someone feels, I usually know what happened to that person, My behavior is a reflection of my inner feelings.

The dominant parenting style was identified using the tool PSDQ – Short version – Parenting Styles and Dimensions Questionnaire (108). The tool consists of 41 items, there are three parenting styles investigated (authoritative, authoritarian, respectively permissive parenting style), the answers being provided on a five-step Likert scale, where 1 – Never and 5 – Always. Examples of items: I give my child reasons why the rules must be followed, I allow my child to contribute to the family rules, I punish my child by taking away privileges, giving little or no explanation.

The level of parental competence was measured using the tool Parenting Sense of Competence Scale – (109). The questionnaire consists of 17 items, the responses being provided on a six-step Likert scale, where 1 - Strongly disagree and 6 - Strongly agree. Examples of items: Even though parenthood could provide many satisfactions, right now I am a parent with many frustrations, Being a parent is easy to manage, and problems that may arise always have a solution, I meet my own expectations regarding child care.

The level of parents’ self-esteem was measured using the RSES tool - Rosenberg Self-Esteem Scale, Morris (110). The questionnaire consists of 10 items, five being negatively worded, the responses being provided on a four-step Likert scale, where 1 – Strongly agree and 4 – Strongly disagree. Examples of items: I know I have a number of qualities, I wish I had more self-respect, I often tend to feel like a failure.

The Barnum effect found in the specialized literature and known as the Forer effect was measured using the personality questionnaire developed by Bertram Forer (102). The questionnaire consists of 13 items to which 7 items were added that highlight the extent to which the parent is a follower of social networks and non-scientifically validated sources to receive information about the child’s upbringing and education. Answers to all items being provided on a five-step Likert scale, where 1 - To a very large extent and 5 - To a very small extent. Examples The items of this personality test were supplemented with a series of statements about the Barnum effect based on the literature (111). Examples of items: Although you have some personality weaknesses, you are generally able to compensate for them, Disciplined and self-controlled on the outside, you tend to be anxious and insecure on the inside, Security is one of your major goals. These items are part of the questionnaire developed by Bertram Forer (102) that highlights the extent to which respondents experience the Barnum effect. To adapt the questionnaire to the sample and the purpose of the research, 4 more items were added to the 13 items that highlight the extent of experiencing the Barnum effect in parenting. Table 1 lists the 4 tems that were added to the personality questionnaire developed by Bertram Forer (102).

**Table 1:** Analysis of the distribution of responses.

Forums, parenting groups are a safe space to keep abreast of new trends in parenting	Parents’ educational level				
	Secondary studies	High-school studies	Post-secondary studies	University studies	Postgraduate studies
To a very small extent	2	31	10	62	45
To a small extent	4	28	15	87	62
To a moderate extent	4	25	10	85	64
To a large extent	2	12	5	34	11
To a very large extent	0	6	0	3	3
As long as the information is relevant to my child’s situation, it does not matter who wrote it or shared it	Parents’ educational level				
	Secondary studies	High-school studies	Post-secondary studies	University studies	Postgraduate studies
To a very small extent	2	33	17	77	62
To a small extent	6	20	13	87	52
To a moderate extent	2	24	7	71	52
To a large extent	2	20	5	29	15
To a very large extent	0	5	1	7	4
If I attended a parenting course, I would be more interested in the topics discussed than the professional training of the person giving the course	Parents’ educational level				
	Secondary studies	High-school studies	Post-secondary studies	University studies	Postgraduate studies
To a very small extent	2	9	9	53	37
To a small extent	3	22	5	71	43
To a moderate extent	4	37	11	85	59
To a large extent	3	22	11	49	39
To a very large extent	0	12	4	13	7
It is more important to want to know more about the child’s education than the source of information	Parents’ educational level				
	Secondary studies	High-school studies	Post-secondary studies	University studies	Postgraduate studies
To a very small extent	2	19	11	49	36
To a small extent	2	15	5	71	55
To a moderate extent	3	30	5	71	55
To a large extent	5	24	13	56	31
To a very large extent	0	14	6	24	8

In the case of ESCQ-45, PSDQ, the Parenting Sense of Competence Scale, RSES, and the Forer’s personality questionnaire, they were translated into Romanian through retroversion, a method that has been used in other research (see, for example (112),). For each tool, the score was obtained by summing the responses to all the items. In the case of the RSES instrument there were 5 reverse-scored items.The reliability coefficients (Cronbach’s Alpha coefficients - α) in the present study were: α = 0.90 (ESCQ-45), α = 0.75 (PSDQ), α = 0.77 (PSOC), α = 0.86 (Rosenberg Self-Esteem Scale), respectively α = 0.74 (Forer’s questionnaire).

### 3.3. Analysis plan

This research is part of the quantitative paradigm, based on objectivism, while pursuing a precise analysis of the data collected from the respondents.

The research variables were parents’ emotional intelligence, parenting style, parental competence, parents’ self-esteem, the Barnum effect, and parents’ educational level. Data analysis involves a series of statistical operations. Along with descriptive statistics used to measure specific parameters, the following operations were used: distribution analysis, correlation analysis, linear regression, mediation analysis, Anova analysis of variance, confirmatory factor analysis.

## 4. Results

The results were interpreted based on the collected data from a sample of 610 responses. The distribution of the sample by gender, age-range, and educational level can be viewed in Table 2.

**Table 2:** Frequency of respondents according to gender, age and educational level.

	Level	Count	Total	Proportion
Gender	Female	530	610	0.87
	Male	80	610	0.13
Age-range	Under 20 years	6	610	0.01
	21–30 years old	67	610	0.11
	31–40 years old	205	610	0.34
	41–50 years old	216	610	0.35
	Over 50 years old	116	610	0.19
Educational level	Secondary studies	12	610	0.02
	High-school studies	102	610	0.17
	Post-secondary studies	40	610	0.07
	University studies	271	610	0.44
	Postgraduate studies	185	610	0.30

H_a_ is proportion ≠ 0.5.

The means, standard deviations, standard error, minimum, maximum, skewness, kurtosis for each variable are presented in Table 3. The value of skewness varies between - 0.97 and 0.88, and the value of kurtosis varies between - 0.33 and 1.40. Both ranges of values are statistically acceptable. Therefore, after the normality of the research variables was analyzed, all skewness and kurtosis values were within an acceptable range.

**Table 3:** Descriptive analysis of research variables.

	Emotional intelligence	Parental competence	Authoritative parenting style	Authoritarian parenting style	Permissive parenting style	Self-esteem	Barnum effect
N	610	610	610	610	610	610	610
Missing	0	0	0	0	0	0	0
Mean	174.48	72.58	64.60	20.98	35.64	15.63	54.82
Median	176.00	71.00	66.00	20.00	36.00	16.00	55.00
Standard deviation	16.33	10.07	6.91	6.23	6.16	1.59	8.40
Minimum	106.00	43	30	12	18	11	24
Maximum	221.00	100	75	51	54	23	84
Skewness	−0.42	0.20	−0.97	0.88	0.10	0.30	−0.16
Std. error skewness	0.10	0.10	0.10	0.10	0.10	0.10	0.10
Kurtosis	0.51	−0.33	1.40	0.91	−0.07	1.01	0.58
Std. error kurtosis	0.20	0.20	0.20	0.20	0.20	0.20	0.20

### 4.1. Correlational and regression analysis between the level of parents’ emotional intelligence and the level of parental competence

In order to test the association between parents’ emotional intelligence and parental competence, a correlational analysis was carried out between the scores obtained on the questionnaire to measure the level of parental competence and the scores obtained on the three subscales of emotional intelligence. Following this analysis, it was demonstrated that between the two variables: parental competence and emotional intelligence divided into the three subscales, there are positive, statistically significant correlations as follows: parental competence and the subscale Perceiving and understanding emotions (*r* = 0.31, *p* < 0.001), parental competence and the subscale Expressing and labeling emotions (*r* = 0.33, *p* < 0.001), parental competence and the subscale Managing and regulating emotions (*r* = 0.30, *p* < 0.001; Table 4). Following the correlation analysis, it was demonstrated that the hypothesis according to which as the level of emotional intelligence of parents increases, parental competence develops supported by the data.

**Table 4:** Correlational analysis between emotional intelligence scores and parenting competence scores.

		Parental competence	Perceiving and understanding emotions	Expressing and labeling emotions	Emotion management and regulation
Parental competence	Pearson’s r	—			
	*p*-value	—			
Perceiving and understanding emotions	Pearson’s r	0.31	—		
	*p*-value	< 0.001	—		
Expressing and labeling emotions	Pearson’s r	0.33	0.56	—	
	*p*-value	< 0.001	< 0.001	—	
Emotion management and regulation	Pearson’s r	0.30	0.48	0.52	—
	*p*-value	< 0.001	< 0.001	< 0.001	—

This association highlighted the usefulness of specific skills and components of emotional intelligence for raising and educating the child. The better the parent is able to understand, express and manage their own emotions, the more effectively they will be able to manage the entire parenting process.

In order to determine how the level of parental competence varies according to the level of emotional intelligence of the parent, the linear regression was performed between the dependent variable parental competence and the independent variable emotional intelligence. The regression coefficient R, which represents a correlation coefficient with a value of 0.38 (R = 0.38) indicated a reasonable association between the two variables, statistically significant (*p* < 0.001). The value of the regression coefficient R^2^ = 0.15 indicated that 15% of the variability of parental competence is due to the level of emotional intelligence of the parent. The overall regression was statistically significant [R^2^ = [0.15], *F* (1, 608) = [103.97], *p* = [<0.001]; Table 5].

**Table 5:** Linear regression between the parental competence variable and the emotional intelligence variable.

			Overall Model Test			
Model	R	R^2^	F	df1	df2	*p*
1	0.38	0.15	103.97	1	608	< 0.001
Model Coefficients - Parental competence						
Predictor			Estimate	SE	t	*p*
Intercept			31.47	4.05	7.77	< 0.001
Emotional intelligence			0.24	0.02	10.20	< 0.001

Following the correlational analysis and the linear regression, it was demonstrated that the hypothesis according to which as the level of emotional intelligence of parents increases, parental competence develops supported by the data.

### 4.2. Correlational and regression analysis between parents’ self-esteem and parenting competence

Based on the correlational analysis between the parental competence variable and the self-esteem variable, a positive, statistically significant association was obtained between the two variables (*r* = 0.24, *p* < 0.001), which indicates that as the parents’ self-esteem increased, an evolution was also registered in the level of parental competence (Table 6).

**Table 6:** Correlational analysis between the scores obtained on parental competence and those obtained on self-esteem.

		Parental competence	Self esteem
Parental competence	Pearson’s r	—	
	*p*-value	—	
Self esteem	Pearson’s r	0.24	—
	*p*-value	< 0.001	—

In order to determine how the level of parental competence varies according to the parents’ self-esteem, a linear regression was performed between the dependent variable parental competence and the independent variable self-esteem. The regression coefficient R with a value of 0.24 (R = 0.24) indicated a statistically significant association between the two variables (*p* < 0.001). The value of the coefficient R^2^ = 0.06 demonstrated that 6% of the level of parental competence varies according to the level of parents’ self-esteem. The overall regression was statistically significant [R^2^ = [0.06], *F* (1, 608) = [36.55], *p* = [<0.001]; Table 7].

**Table 7:** Linear regression between the parental competence variable and the self-esteem variable.

			Overall Model Test			
Model	R	R2	F	df1	df2	*p*
1	0.24	0.06	36.55	1	608	< 0.001
Model Coefficients - Parental competence						
Predictor			Estimate	SE	t	*p*
Intercept			49.07	3.91	12.55	< 0.001
Self-esteem			1.50	0.25	6.05	< 0.001

The data obtained through the regression and correlational analysis confirm the hypothesis that along with the increase in self-esteem of parents, the level of parental competence varies in a positive sense. This is possible due to the fact that parents with high self-esteem manage to cope well with the specific challenges of the parenting process, thus developing their parenting skills and implicitly parenting skills.

### 4.3. Factor analyis for determining parental competence

The purpose of the confirmatory factor analysis was to analyze the factorial model that determines a high level of parental competence. The factorial model was composed of the following factors: parental competence, emotional intelligence, and parental self-esteem.

For a valid analysis of this factorial model, specific indicators of self-esteem with a negative load were removed. In Table 8 it can be read the estimated values of each indicator specific to the variables parental competence, emotional intelligence, self-esteem. The variables on the basis of which the factor analysis was calculated were coded as follows: PC - Parental Competence, EI - Emotional Intelligence, SE - Self esteem. It is also observed that positive correlations are established between all indicators of the three factors (*p* < 0.001). From Table 9 specific to the factor covariances, it was identified that a positive, reasonable correlation was established between the parental competence factor and the emotional intelligence factor (*r* = 0.44, *p* < 0.001), and between the parental competence factor and the parents’ self-esteem factor established a positive, high correlation (*r* = 0.67, *p* < 0.001).

**Table 8:** Confirmatory factor analysis between factors: parenting competence, emotional intelligence, and parental self-esteem.

Factor	Indicator	Estimate	SE	Z	*p*
Parental competence	PC_1	0.36	0.04	8.34	< 0.001
	PC_2	0.86	0.06	14.40	< 0.001
	PC_3	0.88	0.06	14.85	< 0.001
	PC_4	0.82	0.05	15.18	< 0.001
	PC_5	0.56	0.05	10.30	< 0.001
	PC_7	0.39	0.06	6.90	< 0.001
	PC_9	0.86	0.06	15.62	< 0.001
	PC_11	0.40	0.05	8.36	< 0.001
	PC_12	0.56	0.06	10.03	< 0.001
	PC_13	0.34	0.05	6.56	< 0.001
	PC_14	0.56	0.06	9.54	< 0.001
	PC_15	0.49	0.04	11.24	< 0.001
	PC_16	0.79	0.05	14.37	< 0.001
	PC_17	0.34	0.06	5.84	< 0.001
	PC_6	0.20	0.06	3.52	< 0.001
	PC_8	0.20	0.05	3.91	< 0.001
	PC_10	0.19	0.05	3.66	< 0.001
Emotional intelligence	EI_2	0.38	0.04	10.53	< 0.001
	EI_3	0.30	0.04	8.19	< 0.001
	EI_13	0.38	0.03	12.65	< 0.001
	EI_14	0.35	0.03	9.99	< 0.001
	EI_15	0.35	0.03	12.33	< 0.001
	EI_16	0.45	0.04	12.55	< 0.001
	EI_17	0.49	0.03	15.36	< 0.001
	EI_18	0.41	0.03	14.88	< 0.001
	EI_19	0.39	0.03	13.16	< 0.001
	EI_20	0.35	0.03	12.05	< 0.001
	EI_21	0.54	0.03	17.44	< 0.001
	EI_22	0.51	0.03	16.16	< 0.001
	EI_23	0.46	0.03	15.25	< 0.001
	EI_24	0.50	0.03	16.99	< 0.001
	EI_25	0.40	0.03	12.13	< 0.001
	EI_26	0.34	0.03	11.30	< 0.001
	EI_30	0.29	0.03	8.84	< 0.001
	EI_31	0.31	0.04	7.91	< 0.001
	EI_32	0.33	0.03	12.05	< 0.001
	EI_33	0.27	0.02	11.47	< 0.001
	EI_34	0.39	0.03	13.21	< 0.001
	EI_35	0.47	0.04	12.81	< 0.001
	EI_36	0.42	0.03	14.47	< 0.001
	EI_37	0.40	0.03	13.39	< 0.001
	EI_38	0.43	0.03	14.11	< 0.001
	EI_39	0.40	0.03	14.19	< 0.001
	EI_42	0.43	0.03	14.66	< 0.001
	EI_43	0.51	0.03	17.81	< 0.001
	EI_44	0.48	0.03	16.08	< 0.001
	EI_45	0.37	0.03	12.84	< 0.001
	EI_1	0.29	0.04	8.24	< 0.001
	EI_4	0.24	0.03	8.25	< 0.001
	EI_5	0.11	0.04	2.81	0.005
	EI_6	0.22	0.04	5.19	< 0.001
	EI_7	0.26	0.05	5.38	< 0.001
	EI_8	0.24	0.04	5.83	< 0.001
	EI_9	0.24	0.03	7.76	< 0.001
	EI_10	0.02	0.05	0.32	0.751
	EI_11	0.14	0.03	4.43	< 0.001
	EI_12	0.28	0.03	10.66	< 0.001
	EI_27	0.25	0.04	7.23	< 0.001
	EI_28	0.17	0.04	4.54	< 0.001
	EI_29	0.26	0.03	8.72	< 0.001
	EI_40	0.26	0.03	8.78	< 0.001
	EI_41	0.34	0.04	7.60	< 0.001
Self esteem	SE_4	0.29	0.02	11.81	< 0.001
	SE_5	0.45	0.03	14.67	< 0.001
	SE_7	0.34	0.03	13.38	< 0.001
	SE_8	0.36	0.04	9.52	< 0.001
	SE_9	0.54	0.03	17.18	< 0.001

**Table 9:** Factor covariance: parenting competence, emotional intelligence, parental self-esteem.

		Estimate	SE	Z	*p*
Parental competence	Parental competence	1.00ᵃ			
	Emotional intelligence	0.44	0.04	11.39	< 0.001
	Self esteem	0.67	0.04	19.13	< 0.001
Emotional intelligence	Emotional intelligence	1.00ᵃ			
	Self esteem	0.42	0.04	10.11	< 0.001
Self esteem	Self esteem	1.00ᵃ			

Starting from the confirmatory factor analysis, the hypothesis according to which parental competence is determined by factors such as parents’ emotional intelligence and self-esteem supported by the data. As parental emotional intelligence increases and self-esteem levels are high parental competence develops. Emotional intelligence together with self-esteem contributes to the development of interpersonal intelligence, respectively intrapersonal intelligence, which favors the parent’s relationship with his child in a competent way based on an authoritative style and well-established parenting skills (113).

### 4.4. Mediation analysis of parental self-esteem on the relationship between emotional intelligence and parenting competence

In order to test the hypothesis that the relationship between emotional intelligence and parental competence is mediated by the level of parents’ self-esteem, a mediation analysis was calculated through which was identified the causal relationship between the emotional intelligence of the parent, which represents the independent variable, IV and parental competence representing the dependent variable, DV, but also the causal effect of emotional intelligence on the mediator, M, represented by the parent’s self-esteem. A series of regression analyzes were calculated to test the aforementioned hypothesis.

Results indicated that parents’ emotional intelligence predicted parental competence level *p* < 0.001, d = 0.24, 95% CI [0.19, 0.28]. Analyzing the indirect effect, self-esteem significantly mediated the relationship between parents’emotional intelligence and parental competence *p* < 0.001, d = 0.02, 95% CI [0.01, 0.03] (Table 10).

**Table 10:** Estimated values of self esteem mediator on the relationship between emotional intelligence and parental competence.

		95% Confidence Interval					
Effect	Estimate	SE	Lower	Upper	Z	*p*	% Mediation
Indirect	0.02	0.01	0.01	0.03	3.32	< 0.001	8.62
Direct	0.22	0.02	0.17	0.26	9.31	< 0.001	91.38
Total	0.24	0.02	0.19	0.28	10.21	< 0.001	100.00

Emotional intelligence has a positive effect on self-esteem *p* < 0.001, d = 0.02, 95% CI [0.01, 0.03] and self-esteem has a positive effect on the level of parental competence *p* < 0.001, d = 1.08, 95% CI [0.61, 1.54] (Table 11).

**Table 11:** Mediation analysis of parental self-esteem on the relationship between emotional intelligence and parenting competence.

			95% Confidence Interval				
		Estimate	SE	Lower	Upper	Z	*p*
Emotional intelligence	Self-esteem	0.02	0.00	0.01	0.03	4.86	< 0.001
Self-esteem	Parental competence	1.08	0.24	0.61	1.54	4.55	< 0.001
Emotional intelligence	Parental competence	0.22	0.02	0.17	0.26	9.31	< 0.001

**Figure 1:**
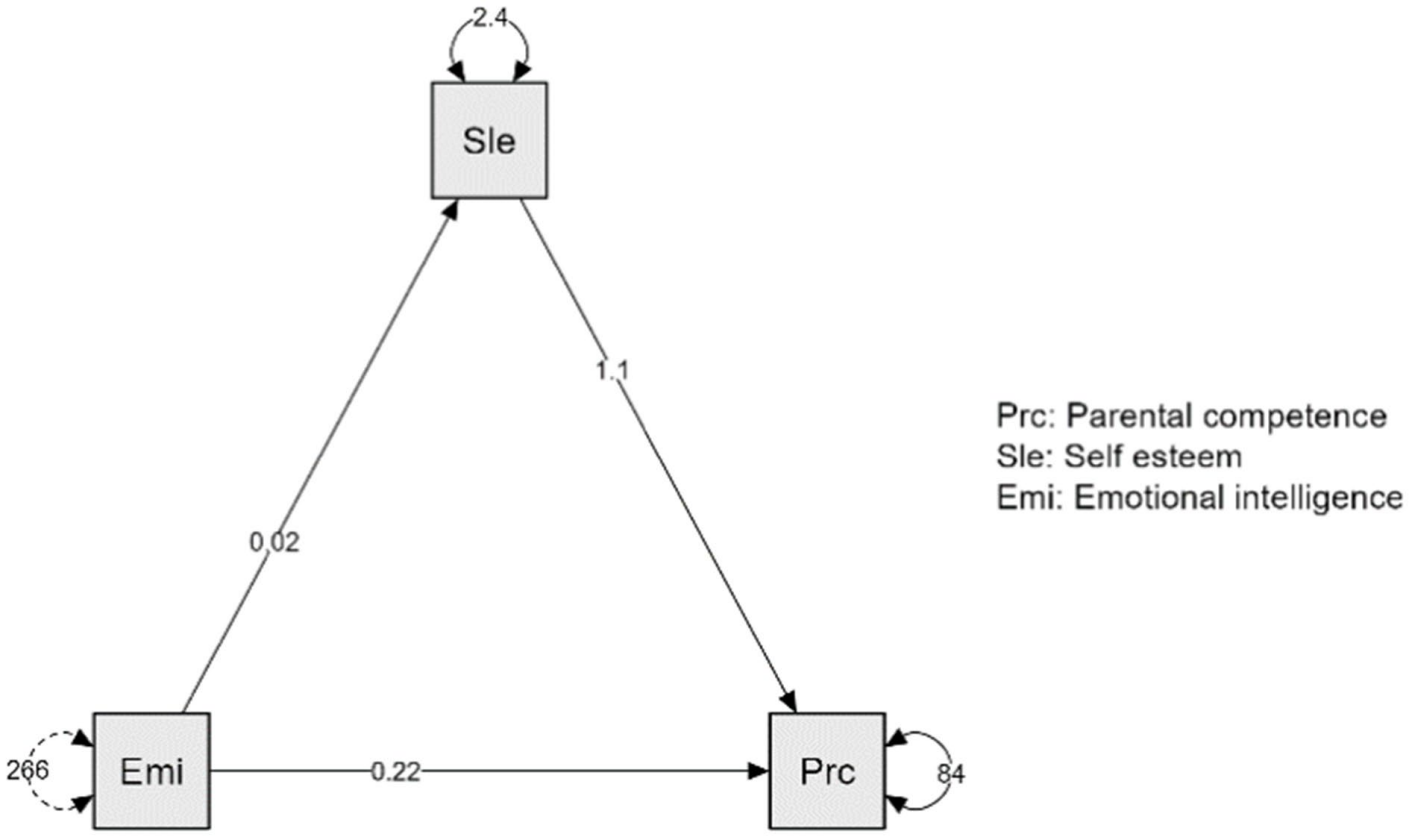
Figure of the mediation analysis.

However, the results suggested that even after taking into account the mediating role of parent self-esteem, parent emotional intelligence had a positive effect on parental competence *p* < 0.001, d = 0.22, 95% CI [0.17, 0.26] (Table 11). As the indirect and direct effect are statistically significant the mediation was partial. The level of parents’ self-esteem represented 8.62% of the total effect of the emotional intelligence of the parent on the level of parental competence, while the emotional intelligence of the parent determined a high level of parental competence in proportion to 91.38% (Table 10). Using Jasp program, the graph of the previously presented mediation model was made (Figure 1).

These results demonstrated that the hypothesis according to which the relationship between emotional intelligence and parental competence is mediated by the level of parents’ self-esteem supported by the data. As the parent demonstrates an increased level of self-esteem, increases the possibility that his emotional intelligence will determine an increased level of parenting competence.

### 4.5. Anova analysis of variance between parental competence and parents’ educational level

An Anova analysis of variance was conducted to determine if there was a significant difference between the level of parental competence based on the educational level of the parents. It was hypothesized that high educational level is associated with a high level of parental competence. The results showed that the level of parental competence differ significantly according to the educational level of the respondents (df = 4 F = 3.68, *p* = 0.009, *p* < 0.05; Table 12).

**Table 12:** Analysis of variance of the level of parental competence according to the educational level of the respondents.

	F	df1	df2	*p*
Parental competence	3.68	4	66.88	0.009
Homogeneity of variance (Levene’s) test of the level of parental competence according to the educational level of the respondents				
	F	df1	df2	*p*
Parental competence	2.57	4	605	0.037

According to the Shapiro–Wilk normality test, this condition is not met for the analysis of variance of parental competence according to the educational level of the respondents, W = 0.99, *p* = 0.005, *p* < 0.05 (Table 12).

The homogeneity of the variance of the level of parental competence according to the educational level is not met, *F* = 2.57, *p* = 0.03, *p* < 0.05 (Table 12).

The Tukey Post-Hoc Test was used to analyze the dependent variable, parental competence. The result of *post hoc* showed that there are statistically significant differences in the level of parental competence depending on the educational level of the respondents in the case of respondents with high school and postgraduate studies, *p* = 0.010, *p* < 0.05 (Table 13).

**Table 13:** Tukey post-hoc test – parental competence.

		Secondary studies	High school studies	Post-secondary studies	University studies	Postgraduate studies
Secondary studies	Mean difference	—	−1.13	−2.28	−3.79	−5.14
	*p*-value	—	0.996	0.958	0.701	0.418
High school studies	Mean difference		—	−1.15	−2.66	−4.01
	*p*-value		—	0.973	0.149	0.010
Post-secondary studies	Mean difference			—	−1.51	−2.87
	*p*-value			—	0.900	0.470
University studies	Mean difference				—	−1.35
	*p*-value				—	0.614
Postgraduate studies	Mean difference					—
	*p*-value					—

* *p* < 0.05.

** *p* < 0.01.

*** *p* < 0.001.

Despite the fact that the conditions of normality and homogeneity were not met, the values of the coefficients of skewness and kurtosis were analyzed. Since the values of these coefficients fall between −1 and +1 (Table 14) it can be stated that the hypothesis according to which the high educational level is associated with a high level of parental competence is partially confirmed.

**Table 14:** Descriptive analysis of the Skewness and Kurtosis values of the level of parental competence depending on the educational level of the parents.

	Educational level	Parental competence
Number of respondents	Secondary studies	12
	High school studies	102
	Post-secondary studies	40
	University studies	271
	Postgraduate studies	185
Skewness	Secondary studies	0.28
	High school studies	0.35
	Post-secondary studies	0.39
	University studies	0.31
	Postgraduate studies	−0.14
Kurtosis	Secondary studies	−0.25
	High school studies	−0.31
	Post-secondary studies	−0.20
	University studies	−0.44
	Postgraduate studies	−0.16

### 4.6. Correlational analysis between parents’ emotional intelligence and authoritative parenting style

By calculating the average of the answers specific to the three subscales, it was found that the central tendency at the sample level is to choose the answer variants that determine an average, respectively high level of emotional intelligence.

The mean of the variable perceiving and understanding emotions is 57.83, the variable expressing and labeling emotions is 54.53 and the variable managing and regulating emotions is 62.13 (Table 15). The value of skewness varies between – 0.70 and – 0.28, and the value of kurtosis varies between – 0.15 and 1.67 Both ranges of values are statistically acceptable. Therefore, after the normality of the research variables was analyzed, all skewness and kurtosis values were within an acceptable range.

**Table 15:** Mean of responses to the questionnaire to measure the level of emotional intelligence.

	Perceiving and understanding emotions	Expressing and labeling emotions	Management and Regulation of emotions
N	610	610	610
Missing	0	0	0
Mean	57.78	54.53	62.13
Median	59.00	55.00	62.00
Standard deviation	7.15	6.81	5.89
Minimum	35.00	18	43
Maximum	75.00	70	79
Skewness	−0.38	−0.70	−0.28
Std. error skewness	0.10	0.10	0.10
Kurtosis	0.21	1.67	0.15
Std. error kurtosis	0.20	0.20	0.20
Shapiro–Wilk W	0.99	0.97	0.99
Shapiro–Wilk p	< 0.001	< 0.001	< 0.001

Based on the descriptive statistics, it was found that the average of the responses to the three subscales of the questionnaire, parenting style authoritarian, authoritative and permissive is higher for the authoritative parenting style, 64.60, followed by the mean of the permissive parenting style, 35.64 and the mean of the authoritarian parenting style, 20.98 (Table 16). The value of skewness varies between – 0.97 and 0.88, and the value of kurtosis varies between – 0.07 and 1.40 Both ranges of values are statistically acceptable. Therefore, after the normality of the research variables was analyzed, all skewness and kurtosis values were within an acceptable range.

**Table 16:** The average of the answers for measuring the variables: authoritative, authoritarian and permissive parenting style.

	Authoritative parenting style	The authoritarian parenting style	Permissive parenting style
N	610	610	610
Missing	0	0	0
Mean	64.60	20.98	35.64
Median	66.00	20.00	36.00
Standard deviation	6.91	6.23	6.16
Minimum	30	12	18
Maximum	75	51	54
Skewness	−0.97	0.88	0.10
Std. error skewness	0.10	0.10	0.10
Kurtosis	1.40	0.91	−0.07
Std. error kurtosis	0.20	0.20	0.20
Shapiro–Wilk W	0.94	0.94	1.00
Shapiro–Wilk p	< 0.001	< 0.001	0.118

Most of the respondents participating in the research are followers of the authoritative parenting style.

Following the correlational analysis between the level of parents’ emotional intelligence and the authoritative parenting style, the existence of a reasonable, statistically significant positive association was found (*r* = 0.45, *p* < 0.001), which indicated that as the level of parents’ emotional intelligence increased, the preference for the authoritative parenting style increased (Table 17). Thus, parents who manage to better perceive their emotions, understand them, express them and later regulate them, tend to find a balance between authority and gentleness, flexibility shown towards the child.

**Table 17:** Correlational analysis between the emotional intelligence total score variable and the authoritative style score variable.

		Emotional intelligence	Authoritative parenting style
Emotional intelligence	Pearson’s r	—	
	*p*-value	—	
Authoritative parenting style	Pearson’s r	0.45	—
	*p*-value	< 0.001	—

Based on the statistical analyses, it was demonstrated that the hypothesis according to which as the level of parents’ emotional intelligence increases, the preference for the authoritative parenting style supported by the data.

### 4.7. Correlational analysis between the Barnum effect experienced by parents and the level of parental competence

Before testing the hypothesis that considers the experience of the Barnum effect in the case of parents depending on their educational level, we present the distribution of the response options to the items specific to the measurement of this effect was calculated in relation to the educational level of the parents. The questions subjected to the distribution analysis were the following: Forums, groups of parents constitute a safe space to be aware of new trends in parenting; As long as the information fits my child’s situation, it does not matter who wrote it or shared it; If I were to attend a parenting course, I would be more interested in the topics discussed than the professional training of the person giving the course; It is more important to want to know more about the child’s education than the source of information (Table 1).

Following the analysis of the data distribution, the following answers were identified by the respondents who graduated from university, respectively post-university studies: forums, groups of parents were considered by 200 respondents to a moderate extent, respectively to a large and very large extent a safe space for to be aware of the new trends in parenting, while 256 respondents considered to a very small or a small extent that these virtual spaces are safe for valid information about parenting.

Of the 456 respondents, 178 considered to a moderate extent, respectively to a large and a very large extent that as long as the information read is suitable for their own child’s situation, it is not important who wrote or spread that information, while 278 respondents they agreed with this statement very little, respectively to a small extent.

252 respondents out of the 456 stated that they are moderately, highly and very interested in the topics discussed in a parenting course and not necessarily in the professional training of the person giving the course. At the opposite pole are the 204 respondents out of a total of 456 who stated that they are interested to a very small or small extent in the topics discussed at the expense of the attention given to the professional training of the person who provides the content of the parenting topics.

It was found that the parent’s desire to know information about the child’s education is more important than the source of information, 245 of them affirming that they agree to a moderate extent, respectively to a great extent and to a very great extent with the previously stated statement, while 211 respondents from the 456 pay more attention to the knowledge of the information at the disadvantage of the information source to a very small or small extent.

Next, despite the high educational level, parents tend to be guided by the topics discussed in a parenting course without paying much attention to the professional training of the person providing these topics. The tendency to search for information about child education without taking into account the possibility that the documentation source may not have scientific content specific to the field was also highlighted.

To test the hypothesis that takes into account the fact that as the parent experiences a high level of the Barnum effect, the level of parental competence decreases, a correlation analysis was carried out between the independent variable the Barnum effect and the dependent variable parental competence. Following this analysis, a negative association was obtained between the two variables *r* = − 0.19, *p* < 0.001. Thus, it was found that as the level of the Barnum effect increases, an effect according to which parents do not choose the right parenting practices from scientific sources or from specialized literature, the level of parental competence decreases, the hypothesis being supported by the data (Table 18).

**Table 18:** Correlational analysis between the Barnum effect experienced by parents and the level of parental competence.

		Barnum effect	Parental competence
Barnum effect	Pearson’s r	—	
	*p*-value	—	
Parental competence	Pearson’s r	−0.19	—
	*p*-value	< 0.001	—

## 5. Discussion

The purpose of the research is to provide scientific information regarding the influence of emotional intelligence on parenting style, respectively on parental competence. A second aim of the research is represented by the identification of the factors that determine the level of parental competence of the parents. The educational level of the parents becomes an important variable of the study being put in relation to both the parental competence and the Barnum effect experienced by the parents. The collected data supported a number of seven hypotheses that are summarized in Table 19.

**Table 19:** Hypothesis testing summary.

Code	Hypothesis short description	*p*-value	Result
H1	EI → PC	<0.001	Supported
H2	PSE → PC	<0.001	Supported
H3	PC → EI + PSE	<0.001	Supported
H4	PSE → EI + PC	<0.001	Supported
H5	PC → EL	<0.05	Partially confirmed
H6	EI → APS	<0.001	Supported
H7	BE → PC	<0.001	Supported

The variable codes are as follows: EI, emotional intelligence; PC, parental competence; PSE, parental self esteem, EL, educational level, APS, authoritative parenting style, BE, Barnum effect.

The results obtained through the statistical interpretation of the data highlight the usefulness of parents’ emotional intelligence and their level of self-esteem for the acquisition of parental competence. The research contributes with novel elements in the field of parenting by determining a factorial model, but also by carrying out a mediation analysis of parents’ self-esteem on the relationship between their emotional intelligence and parental competence. The measurement of the Barnum effect, according to which parents consider true information about the education of children, is not scientifically validated, represents another element of originality of this work.

The positive relationship between parents and children is of great importance for children’s health, well-being and resilience, and the emotional intelligence of parents largely determines this positive relationship (114). Both the process by which children will be taught to behave appropriately and the development of their sense of security depend on the quality of this relationship, which subsequently leads them to have a solid self-perception, but also self-confidence, thus exploiting their potential (115).

Raising children is also accompanied by stressful situations caused by the uncertainty of parents to act in different contexts. Emotional intelligence, along with its skills and competencies, self-knowledge, awareness and management of one’s emotions, self-evaluation and self-control, can contribute to managing stressful situations and adopting constructive thinking in the case of parents (116). Throughout the parental experience, the emotional state of adults can be accentuated as a result of the multiple challenges encountered in the process of educating children (117), which is why emotional intelligence becomes important in regulating the emotional state of parents.

Even if the adult has proven that he has the necessary skills to educate his child, when faced with stressful, confusing times, parenting skills can be affected. Parental competence is correlated with the parent’s feelings of safety and protection in order to face well the challenges that may arise in the education of the child (118), but also with the observance of consequences among children for their inappropriate behaviors at the expense of the application of punishments (119). This is supported by behaviors such as: adaptation to the child’s needs, but also to the contexts that require the formulation of limits; avoiding orders, ultimatums, but adopting a firm but at the same time benevolent attitude that leads to cooperation; spending time with children to strengthen the relationship (120). Parental competence is also proven when parents choose to change their perspective on the problems that may appear in their children’s lives, understand their way of acting and finally offer the right answers to make the problem-solving process more efficient (121).

One of the goals of the competent parent is to demonstrate to the child that he is genuine and sincere when he wants to understand his concerns or perspectives on certain things (122). Another goal of the competent parent is to identify what their child’s abilities, preferences, beliefs, or goals are and act accordingly (123).I.t has been demonstrated that the choice of a profession corresponding to the level of education determines the formation of parental skills through which parental competence is formed (124). Along with parental competence, parenting style is an important aspect for acquiring this competence (125). carry out a differentiated analysis of the three concepts specific to parenting: parenting practices, parenting dimensions, parenting styles. Parenting practices are the observable behaviors of parents for children’s development. For example, a parenting practice that supports children’s academic success could be represented by occasional participation in documenting activities in partnership with children. Positive reinforcement, discipline or conflict management are also included in the category of parenting practices.

Along with parental practices, in the field of parenting there is also the concept of parental dimensions that can be observed in the establishment of relationships between parents and children. The parental dimensions are as follows: Parental support – denotes a parent–child relationship based on affectivity, involvement, acceptance, emotional availability, warmth and responsiveness [(126) cited in (125)]. This dimension was associated with the children’s avoidance of dangerous environments, vices, but also with the externalization of emotions; Parental control – highlights how the parent manages the child’s behavior using rules, disciplinary strategies, punishments and rewards, or supervision. When parents find a balance in controlling the child’s behavior, its development is positively influenced, while inconsistent control (associated with the permissive approach) and exaggerated control (physical punishments, emotional abuse) are associated with inappropriate child behaviors that cause gaps in its development. Parental psychological control - manifests itself through the parent’s attempt to manipulate the child’s thoughts, emotions and feelings [(127, 128) cited in (125)] being associated with relationship difficulties, but also with depressive states.

Self-esteem develops as a result of the experiences that a person lives throughout his life, but also as a result of deep self-reflections on these experiences (129). For a better understanding of the dynamics of self-esteem it is necessary to make a distinction between self-esteem as a trait of the parent and self-esteem as a state of the parent. Self-esteem as a state of the individual represents the totality of his feelings about himself that fluctuate also under the influence of external factors, while self-esteem as a trait refers to the individual’s general assessment of his own worth (130). Parents’ self-esteem is translated into their overall opinion of themselves, reflecting how they feel about their abilities and limitations, as opposed to self-image, which highlights how parents perceive themselves in terms of how they show or opinion of who and what they are. Both aspects are particularly important: a negative self-image affects self-esteem and self-confidence (131), but an optimal level of self-esteem indicates the balanced development of internal comfort and security (132).

A differentiation between parent self-esteem and self-compassion is necessary to establish balance. Self-esteem tends to increase when the parent experiences success in various areas of his life, while self-compassion emphasizes how the parent relates to himself in situations where he experiences failure. Self-esteem needs to be counterbalanced by self-compassion in order for the parent to realize that his human value does not necessarily lie in the success and failure experienced over time, but rather in how he capitalized on successes and handled failures (133).

The limit of the study is represented by a low level of heterogeneity, the majority of respondents being female. As a result of this limitation, hypotheses regarding the role of gender as a moderator of the relationship between emotional intelligence and parenting competence, or the relationship between emotional intelligence and parenting style, could not be tested. In the future, this problem could be solved by choosing a more efficient sampling strategy, which would contribute to the collection of data from more male respondents.

## 6. Conclusion

As a result of the tested hypotheses, the following aspects are concluded:

Parents with a high level of emotional intelligence demonstrate a high level of parental competence as a result of the specific emotional intelligence strategies they use in the parenting process.

The level of parental competence varies with parental self-esteem by 6% as a result of the self-confidence and effectiveness of parents with high levels of self-esteem.

Parental competence is determined by factors such as the emotional intelligence and self-esteem of parents, a confirmatory factor analysis being carried out in this regard. At the same time, self-esteem plays a mediating role in the relationship between parents’ emotional intelligence and parenting competence.

The level of education of the parents partially influences the level of parental competence, additional measures being necessary to formulate a conclusion.

The high level of emotional intelligence of parents is associated with the adoption of the authoritative parenting style that is in agreement with specific elements of this intelligence.

Even if the high educational level of the parents would ensure them access to valid information from a scientific point of view, the Barnum effect is also felt in the case of parents with university and postgraduate studies, which draws attention to the training of adults in terms of parental education regardless of their status or educational level.

As a result of the Barnum effect experienced by the participants of this study, it is noted that the level of parental competence decreases as the Barnum effect increases. Thus, the necessity of training adults in the field of parenting is found once again in order to reduce the adoption of inappropriate educational practices for the education of children.

## Data availability statement

The original contributions presented in the study are included in the article/supplementary material, further inquiries can be directed to the corresponding author.

## Ethics statement

This study was conducted in accordance with the Helsinki Declaration and approved by the Ethics Commission of the University of Bucharest–Registration number: (100/22.03.2022). The patients/participants provided their written informed consent to participate in this study.

## Author contributions

AȘ and GP: conceptualization, methodology, validation, writing – original draft preparation, and writing – review and editing. GP: supervision. All authors contributed to the article and approved the submitted version.

## Conflict of interest

The authors declare that the research was conducted in the absence of any commercial or financial relationships that could be construed as a potential conflict of interest.

## Publisher’s note

All claims expressed in this article are solely those of the authors and do not necessarily represent those of their affiliated organizations, or those of the publisher, the editors and the reviewers. Any product that may be evaluated in this article, or claim that may be made by its manufacturer, is not guaranteed or endorsed by the publisher.
